# Application of ultrasound imaging biomarkers (HistoScanning™) improves staging reliability of prostate biopsies

**DOI:** 10.1186/s13104-017-2896-y

**Published:** 2017-11-09

**Authors:** M. F. Hamann, D. Meyer, S. Knüpfer, J. Fuchs, K. P. Jünemann, C. M. Naumann

**Affiliations:** Department of Urology and Pediatric Urology, University Hospital Schleswig–Holstein, UKSH, Campus Kiel, Arnold Heller Strasse 3, 24105 Kiel, Germany

**Keywords:** Prostate cancer, Prostate biopsy, Ultrasound, Imaging biomarkers, HistoScanning

## Abstract

**Objective:**

Imaging biomarkers like HistoScanning™ augment the informative value of ultrasound. Analogue image-guidance might improve the diagnostic accuracy of prostate biopsies and reduce misclassifications in preoperative staging and grading.

**Results:**

Comparison of 77 image-guided versus 88 systematic prostate biopsies revealed that incorrect staging and Gleason misclassification occurs less frequently in image-guided than in systematic prostate biopsies. Systematic prostate biopsies (4–36 cores, median 12 cores) tended to detect predominantly unilateral tumors (39% sensitivity, 90.9% specificity, 17.5% negative and 50% positive predictive values). Bilateral tumors were diagnosed more frequently by image-guided prostate biopsies (87.9% sensitivity, 72.7% specificity, 50% negative and 96.8% positive predictive values). Regarding the detection of lesions with high Gleason scores ≥ 3 + 4, systematic prostate and image-guided biopsies yielded sensitivity and specificity rates of 66.7% vs 93.5%, 86% vs 64.5%, as well as negative and positive predictive values of 71.2% vs 87%, and 83.3% vs 79.6%, respectively. Potential reason for systematic prostate biopsies missing the correct laterality and the correct Gleason score was a mismatch between the biopsy template and the respective pathological cancer localization. This supports the need for improved detection techniques such as ultrasound imaging biomarkers and image-adapted biopsies.

## Introduction

Therapeutic management of prostate cancer (PCa) commonly relies on clinical staging parameters generated by systematic biopsies. However, a high rate of reclassifications ranging from 28 to 60% in series of active surveillance and radical prostatectomy (RP) has led to increasing criticism of this diagnostic approach [1–4]. State-of-the-art imaging biomarkers in multiparametric magnetic resonance imaging (mpMRI) have recently been shown to improve the diagnostic reliability of prostate biopsies significantly by facilitating the detection and targeting of index lesions [5]. Similarly, Prostate HistoScanning™ (PHS) is an imaging tool that analyses ultrasound biomarkers and potentially enables the surgeon to target cancer-suspicious lesions [6]. Although the technique of PHS has previously shown contradictory results regarding the mere detection of PCa [7–11], its impact on detecting the characteristics of clinically significant tumors remains unclear. Therefore, we retrospectively compared the clinical staging and pathological outcome characteristics in prostatectomy patients from our department, diagnosed either by common transrectal systematic 12 core biopsy (sPB) r vs. image-guided extended perineal PHS biopsy (iPB).

## Main text

### Patient population, data collection, statistical analysis

We searched our institutional database and retrospectively reviewed the clinical characteristics and pathology reports of 231 consecutive patients who had undergone RP at our hospital. All patients (165) with clinical diagnosis of a localized PCa—detected either by sPB, or iPB—were included. Patients with advanced disease, hormonal therapy prior to surgery, or a latency of more than 6 weeks between the detection of PCa and RP were excluded from the study to minimize the bias of cancer progress. We identified a total of 88 patients who underwent sPB and 77 patients who underwent iPB.

iPB was exclusively performed at our clinic. It was planned according to the findings of Prostate HistoScanning™ (PHS, Software version 2.3, Advanced Medical Diagnostics, Waterloo BE). PHS identifies putative locations of malignant tissue by computerized ultrasound data analysis and displays it as a colored overlay in 3D image reconstructions of each prostate. This allows for stereotactic biopsy guidance of millimetric accuracy. But in contrast to the homogeneous sPB template, PHS usually suggests eccentric biopsy placement [10, 11]. This improves iPB at the expense of extended core numbers which ranged from 12 to 18, with a median of 14 (Table 1). sPB was performed at local urologist practices who referred the patient to our hospital for RP afterwards. In the sPB group, the number of cores ranged from 4 to 36, with a median of 12 (Table 1).

**Table 1:** Patient characteristics and biopsy parameters

	Biopsy groups		Overall	*P value*
	Image-guided	Systematic		
No. of patients (%)	77 (46.7)	88 (53.3)	165	
Median age at operation (IQR)	71 (8)	69 (9)	70 (9)	0.127
Median PSA (ng/ml), (IQR)	8 (5.1)	7.9 (6.1)	8 (6)	0.825
Median prostate volume (ml), (IQR)	42 (24)	40 (29)	40 (27)	0.528
Mean No. of cores/biopsy, (± SD)	13.7 (± 1.1)	12.1 (± 4.6)	12.8 (± 3.5)	< 0.001*
Mean Biopsy density (core/ml) (± SD)	0.342 (± 0.19)	0.320 (± 0.19)	0.334 (0.17)	0.066

*IQR* interquartile range (Q3–Q1); *CI* confidence interval; *SD* standard deviation

* Statistical significant difference, Mann–Whitney U Test, (P < 0.05)

RP specimens were sampled following a cross examination protocol that is consistent with the guidelines recommended by the 2009 International Society of Urological Pathology consensus conference [12]. The biopsy and RP specimens were analyzed by multiple pathologists at our institution. Grading discordance was defined as a difference in reporting the presence or absence of high grade (Gleason grade ≥ 4) tumors in biopsy vs. RP specimens. A Gleason upgrade was defined either as the presence of higher Gleason score (GS) in the RP specimen compared to the biopsy GS max, or a shift from GS 3 + 4 to 4 + 3. Vice versa, a shift from 4 + 3 to 3 + 4 or to a lower GS in the final pathology of the RP specimen compared to the previous biopsy was defined as a Gleason downgrade. Anatomic staging of tumors on the basis of their location within the prostate failed due to inconsistent biopsy reporting. Hence we only compared the correct laterality of biopsy reporting, i.e. whether the tumor was found in the left or the right side, or in both sides of the prostate. Prostate volume was measured by ultrasound during the biopsy procedure.

All patients provided a written informed consent for the procedure. Patients were advised that information collected from their biopsies would be used for internal analysis and medical research as approved by the local Ethics Committee (D522/15).

All data were registered in a Microsoft Access database (2010) and subsequently tabulated in Microsoft Excel (2010), with statistical analysis performed using R (Version 3.3.2) and ComKappa 3 by R. Bakeman. The characteristics of both groups were compared using the independent Welch-*t* test, Pearson Chi square and Fisher’s exact tests for categorical variables, and Wilcoxon rank-sum test for non-parametric continuous and ordinal variables. Odds Ratios were computed by conditional maximum likelihood method in R. A P value of ≤ 0.05 was considered to indicate statistical significance.

### Biopsy results, pathological staging and grading characteristics

Overall patient characteristics are given in Table 1. Both biopsy groups demonstrated similar clinical characteristics at diagnosis. Only the total numbers of cores per patients differ significantly in both groups, because image-guided biopsies are usually done in addition to systematic biopsies. Noteworthy in this context are 18 cancers which were detected exclusively by singular targeted iPB biopsies of, while the appendant sextants covered by the systematic backup biopsy were cancer-negative. Therefore, iPB harvested tumor specimen less frequently (34.6%) than sPB (38.9%) when considering the cancer-to-core ratio. However, the biopsy density in terms of cores-per-prostate volume (ml) was similar (Table 1). Pathological findings in RP specimens showed pathological stages ≤ pT2b in 13.3% (22/165) and ≥ pT2c in 86.7% (143/165) of the patients. When stratified by the type of biopsy approach, the quotients of pathological stage ≤ pT2b and ≥ pT2c in sPB and iPB did not differ significantly (Table 2). The pathological GS in RP specimens ranged from 3 + 3 = 6 to 5 + 5 = 10. Again, the proportion of tumors with GS ≤ 3 + 4 and ≥ 4 + 3 in both groups showed no significant differences (Table 2).

**Table 2:** Clinical and pathological outcome characteristics and diagnostic agreement

	Biopsy groups			P value
	Image-guided	Systematic	Odds ratio (95% CI)	
Pathological stage^a^, % (n)			0.86 (0.31–2.34)	.736
≤ pT2b	14.3 (11)	12.5 (11)		
≥ pT2c	85.7 (66)	87.5 (77)		
Clinical stage^a^, % (n)			10.35 (4.60–24.99)	< .001*
≤ cT2b	14.3 (11)	63.6 (56)		
≥ cT2c	85.7 (66)	36.4 (32)		
Stage agreement, % (n)				
Correct	89.6 (69)	46.6 (41)		
Over graded	5.2 (4)	1.1 (1)		
Under graded	5.2 (4)	52.3 (46)		
Cohen’s Kappa (95% CI)	0.58 (0.35 0.80)	0.11 (0.0 0.22)		
Pathological Gleason^a^, % (n)			10.35 (4.60–24.99)	.268
≤ 3 + 4	40.3 (31)	48.9 (43)		
≥ 4 + 3	59.7 (46)	51.1 (45)		
Biopsy Gleason^a^, % (n)			3.36 (1.69–6.84)	< .001*
≤ 3 + 4	29.9 (23)	59.1 (52)		
≥ 4 + 3	70.1 (54)	40.9 (36)		
Gleason score agreement, % (n)				
Correct	81.8 (63)	76.1 (67)		
Over graded	14.3 (11)	6.8 (6)		
Under graded	3.9 (3)	17 (15)		
Cohen’s Kappa (95% CI)	0.61 (0.39 0.82)	0.53 (0.32 0,73)		

*CI* confidence interval

* Statistical significant difference (P < 0.05)

^a^Chi squared test/Fisher’s Exact test

### Clinical staging and grading characteristics

Overall, a significant mismatch between clinical staging and grading characteristics and the final pathological results was evident in both biopsy groups (Table 2). The proportions of cancers in the patient cohort clinically staged ≤ cT2b and ≥ cT2c were 40.6% (67/165) and 59.4% (98/165), and the percentages of tumors with GS ≤ 3 + 4 and ≥ 4 + 3 were 45.5 and 54.5%, respectively.

**Fig. 1:**
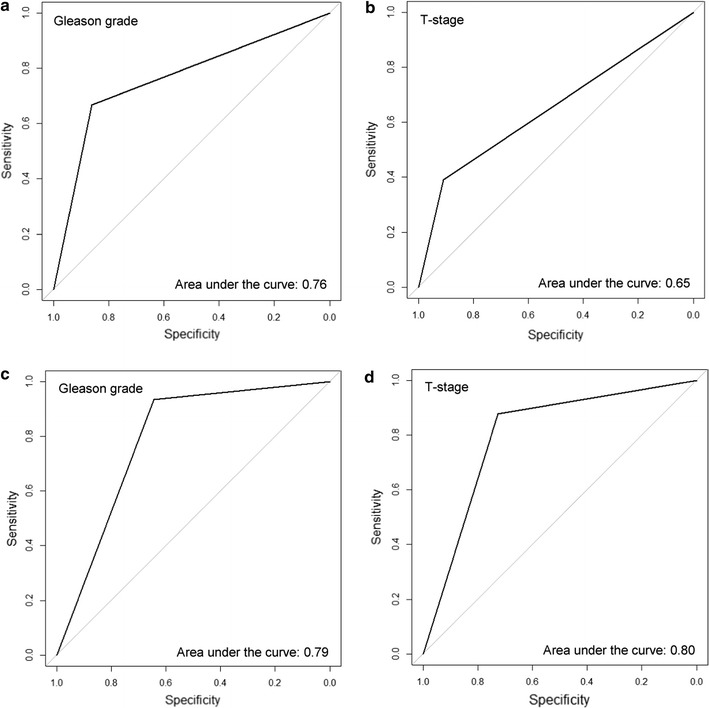
Area under the curve for Gleason grading and staging results per patient. The results from transrectal systematic biopsies are shown in (**a**) and (**b**). The results from perineal image targeted prostate biopsies are shown in (**c**) and (**d**)

SPB tended to diagnose predominantly unilateral tumors, while iPB identified bilateral tumors more often (Table 2). Hence, sPB rarely (1.1%) overstaged the tumor, but instead tended to underestimate the clinical stage in 52.3% (46/88). iPB led to over- and understaging in four patients each (5.2%). This results in an overall staging agreement of 89.6% (69/77) in iPB, but only 46.6% (41/88) in sPB (Table 2). For the detection of bilateral tumors, the sensitivity of sPB and iPB was calculated as 39% (95% CI, 0.280–0.508) and 87.9% (95% CI, 0.775–0.946) respectively. The specificity for these procedure was 90.9% (95% CI, 0.587–0.998) and 72.7% (95% CI, 0.390–0.940), respectively. Bilateral tumors proven in RP specimens were predicted by sPB and iPB with a negative predictive value of 17.5% (95% CI, 0.087–0.299) and 50% (95% CI, 0.247–0.753), and a positive predictive value of 95.1% (95% CI, 0.863–0.990) and 96.8% (95% CI, 0.833–0.999), respectively. Figure 1a and c show the areas under the receiver operating characteristics curves for the sPB and iPB schemes (AUC: 0.65 vs 0.8, *P* = .046).

iPB diagnosed significantly more subjects with high Gleason grade cancers (≥ 4 + 3) than sPB (Table 2). The diagnostic agreement for GS ≤ 3 + 4 in sPB and iPB was 81.8 and 76.1%, respectively. Finally, regarding the prediction of GS ≤ 3 + 4, sPB and iPB had a sensitivity of 66.7% (95% CI, 0.510–0.780) and 93.5% (95% CI, 0.821–0.986), and a specificity of 86%(95% CI, 0.721–0.947) and 64.5% (95% CI, 0.454–0.808), respectively. For prediction of GS ≥ 4+ 3 on RP specimens, sPB and iPB had a negative predictive value of 71.2% (95% CI, 0.569–0.829) and 87%(95% CI, 0.664–0.972) and a positive predictive value of 83.3%(95% CI, 0.672–0.936) and 79.6%(95% CI–0.665; 0.894), respectively. Figure 1 b and d show the areas under the receiver operating characteristics curves for the systematic and targeted biopsy schemes (AUC: 0.76 vs 0.79, *P* = .34).

### Comments

More than one-third of all surgically treated cases of PCa are postoperatively confronted with severely worse tumor characteristics than previously diagnosed [1, 4]. In our series, 17% of the patients from the sPB group had to face upgrading due to final histopathological results. This percentage is in line with existing evidence [13]. To overcome these well-known shortfalls, template biopsy protocols were initially advocated, in addition to novel procedures of imaging biomarkers like mpMRI, or augmented ultrasound technologies. Usually, these modifications entail extended invasiveness due to larger biopsy numbers and therefore conflict with the use of validated risk stratification models [14]. Moreover, the true merit of modern prostate diagnostics should be the acquisition of a representative approach, preferably by guided sampling, without an add-on of additional non-informative biopsies [15]. The percentage of postoperative upgrading through iPB was reduced to 5.2%, which can be seen as a success ultrasound imaging biomarkers which can compensate the lack of selectiveness. It is noteworthy that these results are almost on the same level as mpMRI-TRUS fusion biopsies, which have shown 96% accuracy in detecting the PCa index lesion [16, 17]. Despite the unexcelled diagnostic performance of mpMRI, its adoption is limited due to a lack of availability in hospitals and for economic reasons in most healthcare systems. Ultrasound diagnostics have the potential of providing excellent imaging, with comparatively much less expensive hardware. Therefore, TRUS will probably remain the global standard, not least because of its role in mpMRI fusion procedures. This makes it worthwhile to progress in the field of ultrasound imaging biomarkers [18].

The benefit of PHS in this context is under debate. Most recent studies evaluating the use of PHS attest only minor clinical value because they failed to show a diagnostic advantage over systematic transrectal biopsies [7–9]. In contrast to Porres et al. who reports results in a cohort of 282 patients, rate of the staging and grading congruency in our series shows the efficacy of PHS algorithms in detecting PCa lesions. One might object that iPB characteristics might be attributed to a higher probability of detecting a significant focus “by chance”, simply due to additional biopsies. But, the very similar distribution of core numbers in both groups, measured as biopsy density, renders chance an implausible causal factor, especially when taking into account that 18 cancers were detected by singular targeted biopsies only (Table 1).

We are convinced that the perineal biopsy approach facilitates appropriate sampling. The comparison of perineal and transrectal prostate biopsies sampled in the same patient show significant differences in favor of the perineal approach [10]. Based on geometric evaluation, Han et al. showed that systematic biopsies with freehand TRUS guidance do not closely follow the sextant scheme. A mean targeting error of 9.0 mm and clustered biopsy locations result in suboptimal sampling and cancer detection [19]. Our data support these results and show that image-guided perineal guided biopsy differs significantly from transrectal biopsy, which underestimates the clinical stage in 52.3% of the subjects. These results are also in line with existing evidence [16].

While inferior to the characteristics of mpMRI ultrasound fusion biopsies, our study results still demonstrate the benefit of image-guidance and solid biopsy techniques. Irrespective of the inconsistent evidence on the advantages of PHS, our results question the practice of systematic transrectal biopsy. Further development in the field of intraprostatic targeting is needed, as well as multicenter studies defining the value of ultrasound biomarkers.

## Limitations

Sampling errors and inter-examiner differences regarding biopsy core quality influence the clinical outcome, even if adequate experience in performing TRUS biopsy is assumed. Likewise, the results in our study are biased and may not be representative; i.e. may not reflect the results of urological practices or hospitals. Moreover, sPB specimens were analyzed by pathology institutions outside our hospital, while iPB and all RP specimens were analyzed by pathologists at our institution. This may have led to relevant inter-observer variability or prejudice in case of repeated evaluation. A prospective randomized design with cross evaluation by different examiners would be needed to minimize this bias and to obtain improved results.

## Abbreviations

GS: Gleason score
iPB: image-guided perineal extended Prostate HistoScanning™ biopsy
mpMRI: multiparametric magnetic resonance imaging
PCa: prostate cancer
PHS: Prostate HistoScanning™
RP: radical prostatectomy
sPB: systematic transrectal 12 core biopsy
TRUS: transrectal ultrasound of the prostate
